# A review of radiomics and genomics applications in cancers: the way towards precision medicine

**DOI:** 10.1186/s13014-022-02192-2

**Published:** 2022-12-30

**Authors:** Simin Li, Baosen Zhou

**Affiliations:** Department of Clinical Epidemiology and Center of Evidence-Based Medicine, The First Hospital of China Medical University, Shenyang, 110001 Liaoning People’s Republic of China

**Keywords:** Radiomics, Genomics, Cancer, Machine learning, Evidence-based medicine

## Abstract

The application of radiogenomics in oncology has great prospects in precision medicine. Radiogenomics combines large volumes of radiomic features from medical digital images, genetic data from high-throughput sequencing, and clinical-epidemiological data into mathematical modelling. The amalgamation of radiomics and genomics provides an approach to better study the molecular mechanism of tumour pathogenesis, as well as new evidence-supporting strategies to identify the characteristics of cancer patients, make clinical decisions by predicting prognosis, and improve the development of individualized treatment guidance. In this review, we summarized recent research on radiogenomics applications in solid cancers and presented the challenges impeding the adoption of radiomics in clinical practice. More standard guidelines are required to normalize radiomics into reproducible and convincible analyses and develop it as a mature field.

## Background

Currently, tumour tissue biopsies are the protocol for cancer diagnosis [1]. However, the biopsy method has several significant limitations: it is high-risk, highly invasive, expensive, and difficult to perform in serial tests; it has technical limitations resulting from the tumour location and difficulty in extracting cellular subpopulations; and it is unavailable when tumour cells disseminate to other organs and tissues [2]. These limitations restrict the application of tissue biopsy in cancer screening, diagnosis, treatment, and follow-up.

Fortunately, radiomics, a medical imaging method, holds great promise to address the problem of spatially and temporally heterogeneous solid cancers in a noninvasive way. Radiomics was first presented by Lambin et al. in [3] and involves the high-throughput extraction of image features from large numbers of medical images. Radiomics hypothesizes that additional information not seen by the naked eye could be captured via advanced feature analysis from medical imaging. In detail, genomic and proteomic expression profiles can probably be visualized and expressed according to quantitative analysis of image-based features.

With the success of the Human Genome Project (HGP), the application of genomics in medicine shows strong feasibility and prospects, which has brought an anticipated transformation in the prevention, diagnosis, and treatment of diseases. Projects such as The Cancer Genome Atlas (TCGA) have witnessed tremendous strides towards comprehensively cataloguing human genes and mutations. Some specific ones were found to be capable of serving essentially as ‘drivers’ of oncogenesis, primarily via exome or genome sequencing of large numbers of tumour samples matched with normal tissues [4, 5]. According to the definition from the National Human Genome Research Institute, genomic medicine is an emerging medical discipline in which an individual’s genomic information is taken into account in their clinical care (for example, for diagnostic or therapeutic decision-making, as well as the health effects and policy ramifications of that clinical care) [6]. Radiogenomics is an approach that combines large volumes of radiomic features from medical digital images, genetic data from high-throughput sequencing, and clinical-epidemiological data into mathematical modelling [7]. The amalgamation of radiomics and genomics provides an approach to better study the molecular mechanism of tumour pathogenesis and develop new evidence-supporting strategies. It is beneficial to stratify patients, identify the characteristics of cancer patients, make clinical decisions, and guide treatment by predicting prognosis and assessing clinical outcomes, thus improving the development of precision medicine [8].

## Process of radiomics

Generally, the radiomics workflow consists of the following main steps: imaging data collection, imaging preprocessing, identification and segmentation of the region or volume of interest, feature extraction, feature selection, model establishment, and model validation [9, 10].

### Data collection

The original medical images are necessary for performing radiomics analysis. Computed tomography (CT), magnetic resonance imaging (MRI), and positron emission tomography (PET) have been extensively applied in the field of oncology to assist diagnosis and guide treatment. Images can provide detailed information about tumours’ anatomical and functional features. Medical imaging technology has made tremendous progress in the past, enabling the emergence of radiomics as a promising methodology for solving the complex problems of oncology. The development of medical imaging devices has moved from single-slice CT to multi-slice CT, which allows dynamic radiomics at multiple time points. In addition, dual-energy CT has been explored to improve the identification of tissue composition and density [3]. Diffusion-weighted MRI can reflect tumour density and cellularity, thereby having the capacity to monitor the response to cytotoxic treatment [11].

### Imaging preprocessing

The preprocessing step is crucial to obtain medical images with preferred quality for subsequent analysis [12]. Imaging preprocessing steps generally include image normalization, denoising, bias field correction, imaging interpolation and resampling, motion correction, and imaging thresholding [13].

Image normalization is the process of changing the range of pixel intensity values, which is the foundation of medical image analysis. Conventional normalization approaches are generally used per dataset, such as generalized scale normalization, histogram normalization, and spatial normalization [14, 15]. Recent research proposed a joint normalization function across multiple datasets, which provided realism to the normalized images and improved image segmentation [16]. The crucial task of image denoising is keeping the most significant features of the images while simultaneously removing the nonimportant features. Classical denoising methods involve spatial domain filtering and variational denoising methods. The present transform domain methods, such as Fourier transform, cosine transform, wavelet domain methods [17], and sparse 3D filtering [18], were developed from the initial spatial domain methods [19]. Image resampling is divided into upsampling and downsampling. According to the Image Biomarker Standardization Initiative (IBSI), data from different modalities may fit different methods for image interpolation [20]. Motion correction is an approach to remove the motion artefacts caused by uncertain motion, which is also a vital preprocessing step to obtain reconstructed images with significantly improved quality [21].

### Imaging segmentation

Three primary approaches are used to segment the region of interest (ROI) in two dimensions or the volume of interest (VOI) in three dimensions: manual, semiautomatic, and fully automatic. In comparing these approaches, semiautomatic segmentation is considered optimal [22]. ROIs/VOIs determine the region where radiomic features are extracted and calculated [23]. ROI/VOI segmentation through manual delineation is common in previous research and does not require the use of any sophisticated postprocessing methods or software. However, it is time-consuming and thus hard to use when analysing massive imaging data [24]. In addition, human delineation presents an inevitable bias resulting from different observers that cannot be ignored, leading to a lack of robustness due to intra- and interobserver variations [25]. Semiautomatic delineation uses computer algorithms to segment the ROIs/VOIs but usually needs to be corrected and calibrated manually [26]. Some primary open-source or commercial software could be applied to conduct semiautomatic segmentation, such as 3D Slicer [27], ITK-SNAP [28], LIFE [29], MITK [30], and ImageJ [31]. Fully automatic segmentation is based on the approach of deep learning using artificial neural networks. Deep learning has been universally capitalized in many fields associated with automated image recognition. Convolutional neural networks (CNNs), one of the deep neural networks designed for various computer vision tasks, have been successfully applied in imaging recognition and classification [32, 33]. CNN builds up the mapping directly from the input data and output labels, utilizing a hierarchical network to learn abstract features [34]. The advantages of CNN in radiomics are automatic feature extraction and accurate classification performance [35]. However, this method often requires a large sample size of medical images for deep learning. It is suitable when there is a substantial signal difference between the cancer region and the normal tissues [36].

### Feature extraction

Quantitative radiomics features extracted from segmented ROIs/VOIs can be divided into several major categories, including shape, statistical, and texture features [37]. Shape features mainly include elongation, flatness, sphericity, surface area, surface volume, and voxel volume [38]. Statistical features are the first-order features obtained from a grey-level histogram, which presents the distribution of grey-level intensity values and reveals the tumour’s overall heterogeneity without considering spatial information. Texture features are second-order features that represent the spatial relationships between the voxels from matrices, including the grey-level run-length matrix (GLRLM), grey-level size zone matrix (GLSZM), grey-level co-occurrence matrix (GLCM), neighbourhood grey-tone difference matrix (NGTDM), size of homogeneous greyscale areas, length of greyscale ranges, and greyscale dependence. Feature extraction is commonly conducted using radiomic tools, such as PyRadiomics [39, 40], IBEX [41], MIRP [42], RaCaT [43], and RadiomiCRO [44]. These platforms are based on different languages of Python or MATLAB. However, the lack of standardization in the definition and calculation of radiomic features among these tools has hindered the application of radiomics in clinical practice. The IBSI addressed some of the questions and was thus recommended during feature extraction [45]. The IBSI is composed of a set of 169 standardized radiomics features, which could be verified and calibrated across different radiomics software. It provided standardized image biomarker nomenclature and definitions. Some new tools tend to conform to the IBSI, such as S-IBEX, an adaptation of IBEX to IBSI guidelines [46]. Recent research proposed a novel approach for evaluating the agreement among seven radiomics tools. They found that discrepancies still existed among standardized radiomic programs. Therefore, further efforts are needed to accelerate the use of radiomic models in clinical practice [47].

### Analysis and modelling

The core of the radiomics approach is to establish a predictive model. The modelling process is based on the training data analysis. It is indispensable to apply the model on testing data to evaluate its extrapolation performance, proving the model's precision, fit, and feasibility. Generally, hundreds of radiomics features can be extracted from images. Therefore, feature selection needs to be performed to identify the most valuable traits to avoid overfitting, since the incorporation of redundant features into a model will degrade the performance of model extrapolation. Least absolute shrinkage and selection operator (LASSO) plays a prominent role in selecting informative features [48]. Principal component analysis (PCA) is also one of the preferable methods to reduce the dimensions of radiomics features but preserve their informative content [49]. Machine learning (ML) is a branch of artificial intelligence (AI) in which a machine is trained to learn from data without being explicitly programmed and can make decisions with minimal human intervention [50]. A variety of machine learning approaches are used for constructing radiomics models, such as support vector machine (SVM), random forest (RF), k-nearest neighbours (KNN), decision tree (DT), conditional inference trees (CIT), Bayes and back-propagation neural network (BPNet) [51]. One of the most technical steps is hyperparameter tuning and optimization for the learning algorithm to improve the performance of ML models [52]. The common approaches to tuning the hyperparameters [53] of the models included grid search, repeated stratified k-fold cross-validation, genetic algorithms (GAs), random search [54], sequential search, the Gaussian process approach (GP), tree-structured Parzen estimator (TPE), and Bayesian optimization [55, 56]. Recent research reported that model-based optimization (MBO) could be an excellent tuning strategy for RF. The R package tuneRanger was used to automatically tune RF with MBO [57]. Another study developed a metaheuristic algorithm using grey wolf optimization (GWO) and GA to tune the hyperparameters of ML algorithms and neural networks [58]. Notably, the metaheuristic methods displayed better and faster performance than other algorithms and are appropriate for datasets with unknown distributions. Traditional statistical methods, specifically univariate and multivariate analyses, are used to identify the statistically significant variables and radiomics signatures associated with the outcome [59]. Afterwards, logistic regression and Cox proportional hazards regression are often used to build a radiomics-clinical predictive signature that incorporates clinical risk factors and radiomics features, providing a basis for diagnosis, clinical treatment options, and prognostic prediction [60, 61].

In addition, deep learning provides a non-engineered radiomics modelling methods and has been applied in various medical image analysis tasks, including supervised learning, unsupervised learning, and semi-supervised learning [62]. ﻿Models are trained on labelled images in supervised learning, such as CNNs. In contrast, models in ﻿unsupervised learning analyse the underlying information in the images without any labels, such as ﻿autoencoders (AE) [63] and generative adversarial networks (GANs) [64]. In the ﻿semi-supervised learning, only a part of the training datasets is labelled, and the model will perform better through learning the unlabelled images [65]. Deep learning models based on artificial neural networks such as CNNs can be established to classify target lesions delineated in medical images, such as the discrimination of tumours as benign or malignant. The processes of data learning and classification are performed jointly in the deep learning approach [33]. Existing structures such as VGG [66], Resnet [67] or self-designed networks can be used for CNN. Transfer learning can be performed by tuning hyperparameter depending on the data size, providing a convenient approach to apply the existing deep learning algorithms.

The last step of the radiomics approach is assessing performance and external validation based on other datasets to prove the credibility of the established model. The performance can be evaluated using the receiver operating characteristic (ROC) curve, the area under the ROC curve (AUC), and the C-index to assess the discrimination of the model, and calibration curves can be used to appraise the calibration in the training and validation sets. The model's potential clinical value can be estimated by using decision curve analysis (DCA) [68]. In addition, resampling methods (for instance, cross-validation and bootstrapping) can also be applied to evaluate model performance [69].

## Current applications of radiogenomics in cancer

The literature on radiogenomics has greatly expanded in the past decade. Radiogenomics has been applied in various types of cancer, including lung cancer [70–72], breast cancer [73–75], genitourinary cancer [76–78], digestive system cancer [79–81], and brain cancer [82–84]. The comprehensive analysis method of radiogenomics can be used to diagnose and predict patient outcomes such as recurrence, metastasis, and survival of patients with cancer; predict the tumour microenvironment; identify potential molecular biomarkers and targets for clinical diagnosis and treatment; and establish a molecular typing system of variable cancers. A selection of papers was compiled for illustrative purposes and the characteristics of the representative papers are displayed in Table 1.

**Table 1:** Characteristics of studies on the current applications of radiogenomics in cancer

Research	Cancer type	Application	Sample size	Images	Methods	Features in models	Results
Chen et al. [85]	ccRCC	Diagnosis	197	CT	LR	Radiomics; genomics	AUC: 0.864–0.900
Smedley et al. [86]	NSCLC	Diagnosis	351	CT	NN	Radiomics; genomics	AUC: 0.86 (adenocarcinoma), 0.91 (squamous cell), and 0.71 (other)
Shim et al. [87]	Glioblastoma	Recurrence	192	MRI	NN	Radiomics;	AUC: 0.969 for local recurrence and 0.864 for distant recurrence
Kirienko et al [88]	NSCLC	Recurrence	151	PET/CT	GLM; ML	Radiomics; genomics	AUC: 0.87
Yan et al. [89]	MB	Survival	166	MRI	LASSO-COX	Radiomics; clinicomolecular	C-index: 0.762 for OS and 0.697 for PFS
Xie et al. [81]	ESCC	Survival	106	CT	ML	Radiomics; clinical factors	AUC: 0.852 for 5-year DFS; Significant risk stratification for DFS (p < 0.001)
Huang et al [90]	ccRCC	Survival	205	Contrast-enhanced CT	RF	Radiomic; genomics	AUC: 0.84, 0.81, and 0.75 for 1, 3, and 5-year OS, respectively
Liu et al. [80]	CRC	Metastasis	134	CT	LR	Radiomics; genomics; clinical factors	AUC: 0.752 (95% CI 0.608–0.896)
Kim et al [92]	Paediatric osteosarcoma	Chemotherapy response	73	PET/CT	ML	Radiomics; genomics;	AUC: 0.89
Yi et al. [93]	OC	Platinum resistance	102	CT	SVM	Radiomics; genomics; clinical factors	AUC: 0.967 (95% CI 0.83–0.98)
Zeng et al. [77]	ccRCC	Molecular subtypes	382	Contrast-enhanced CT	ML	Radiomics; genomics; transcriptomics; proteomics	AUC: 0.973 (m1), 0.968 (m2), 0.961 (m3), 0.953 (m4)
Park et al. [94]	Glioblastoma	Molecular characteristics	121	MRI	ML	Radiomics; clinical factors	AUC: 0.863
Li et al. [95]	BC	Molecular subtypes	91	MRI	Linear classifier	Radiomics	AUC: 0.89 (ER + vs. ER −), 0.69 (PR + vs. PR −), 0.65 (HER2 + vs. HER2 −), and 0.67 (triple-negative vs. others)

*ccRCC*: clear cell renal cell carcinoma; *NSCLC*: non-small cell lung cancer; *MB*: medulloblastoma; *ESCC*: oesophageal squamous cell carcinoma; *CRC*: colorectal cancer; *OC*: ovarian cancer; *BC*: breast cancer; *CT*: computed tomography; *MRI*: magnetic resonance imaging; PET: positron emission tomography; *NN*: neural network; *GLM*: generalized linear model; *ML*: machine learning; *LASSO-COX*: least absolute shrinkage and selection operator penalized Cox proportional hazards regression; *RF*: random forest; *LR*: logistic regression; *SVM*: support vector machine; *AUC*: area under the curve; *OS*: overall survival; *PFS*: progression-free survival; *DFS*: disease-free survival

### Diagnostic value

Radiomics predictive models have diagnostic value capable of assisting clinicians in making decisions based on big data evidence. Chen et al. [85] established models to identify clear cell renal cell carcinomas (ccRCCs) and non-clear cell renal cell carcinomas (non-ccRCCs). The combined diagnostic model incorporated texture features extracted from CT images and other non-texture features. They found that the model significantly improved the predictive efficacy of ccRCC, with the AUC values for differentiating the two groups ranging from 0.864–0.900. The results showed a good capability for discriminating ccRCCs from non-ccRCCs. An integrative model combining imaging features and genomic information provides an insightful biological explanation from the molecular perspective for lesions on medical images directly visible to the naked eye. Smedley et al. [86] explored deep feedforward neural networks to identify gene expression profiles in an interpretable way, being capable of anticipating the quantitative radiomic features of CT and histological types (adenocarcinoma, squamous cell, and other) of non-small cell lung cancer (NSCLC). The study enrolled 262 and 89 patients from two public databases for training and testing, respectively. The results suggested that neural networks performed better than other classifiers, classifying histopathological types with AUCs of 0.86 (adenocarcinoma), 0.91 (squamous cell), and 0.71 (other) in the testing cohort. The AUCs for the classification performance of radiomics features ranged from 0.42 to 0.89. Gene sets of cardiac, immune system, and cell development processes were predictive (AUC > 0.70) of certain dissimilar radiomic features. In contrast, tumour necrosis factor (TNF), AKT signalling, and Rho gene sets could forecast the features of tumour textures. The research demonstrated that neural networks could be utilized to map the expression of genes to radiomic characteristics and further to histopathological types in NSCLC, which could explain how the models could recognize predictable genes related to various imaging features or histological types.

### Predicting the patient outcome

#### Recurrence

Cancers can be devastating on account of the high recurrence rate. Recently, Shim et al. [87] proposed two different radiomics-based models through a neural network to predict the relapse pattern in glioblastoma, namely, local and distant recurrences. They used high-dimensional radiomics profiles based on dynamic susceptibility contrast-enhanced perfusion MRI. The AUCs were 0.969 (95% confidence interval: 0.903–1.000) for local recurrence and 0.864 (95% confidence interval: 0.726–0.976) for distant recurrence for patients in the validation cohort. Another recent study conducted by Kirienko et al. [88] evaluated the correlations of information from radiogenomics analyses with histotype and patient outcome in NSCLC using an ML algorithm. The results showed that two radiomics features, standardized uptake value and kurtosis, and the expression levels of TP63, EPHA10, FBN2, and IL1RAP were associated with the histology of NSCLC. They also identified robust PET radiomic features and gene expression profiles capable of predicting recurrence (with AUC = 0.87). Radiogenomics analysis may provide valuable evidence for predicting the histological type, invasiveness, and progression when making clinical decisions for patients with NSCLC.

#### Survival

Based on a radiomics cohort of medulloblastoma (MB) patients with MRI data, Yan et al. [89] constructed a radiomics signature combining the most prognostic radiomics features identified for survival prediction in a training cohort including 83 samples. Another radiogenomics cohort with matched MRI images and RNA sequencing (RNA-seq) data was used to identify nine vital biological pathways significantly related to radiomics characteristics. They also assessed the prognostic prediction value of the genes involved in the identified pathways based on an open-access dataset with RNA-seq and survival data on MB. The results indicated that the signature combining radiomics and clinicomolecular factors predicted overall survival (OS) (C-index of 0.762) and progression-free survival (PFS) (C-index of 0.697) more accurately than the separate radiomics model (C-indexes of 0.649 for OS and 0.593 for PFS) or the clinicomolecular model (C-indexes of 0.725 for OS and 0.691 for PFS). The research demonstrated that the radiomics signature was an independent predictive factor for survival in MB patients. The signature was related to dysregulated pathways and provided additive value over the clinicomolecular factors model. In a recent study of oesophageal squamous cell carcinoma (ESCC), Xie et al. [81] concluded that genomics association was advantageous for selecting meaningful radiomic features to establish CT-based radiomic signatures. This could contribute to predicting prognosis in terms of disease-free survival (DFS), particularly individualized long-term survival. The study involved 106 patients with ESCC who received neoadjuvant chemoradiation (nCRT) at two institutions. Radiomic features were selected according to their correlation with differentially expressed genes (DEGs) identified between relapsed and non-relapsed patients and the appended ML approach. A radiomic nomogram integrating the radiomic characteristics with prognostic clinical factors was established for DFS prediction, which significantly stratified patients into two groups, with high risk and low risk for DFS (p < 0.001) (AUCs for predicting 5-year DFS of training set: 0.912, internal test set: 0.852, and external test set: 0.769). The results showed that the radiomic prediction model utilizing genomics-assisted feature selection performed better in DFS prediction.

Contrast-enhanced CT is also a critical imaging examination method in clinical applications. Huang et al. [90] enrolled 205 patients with ccRCC with accessible contrast-enhanced CT images from The Cancer Imaging Archive (TCIA) database and matched transcriptomic data obtained from the TCGA database. They conducted research aiming to incorporate radiomic features extracted from medical images and corresponding genomics information to predict the OS of ccRCC patients. In the results, four prognosis-related imaging features (PRIFs) were selected from 107 extracted radiomics features by performing LASSO Cox regression and SVM-RFE. Four prognosis-related genes were identified through weighted gene coexpression network analysis (WGCNA). A mixed imaging-genomics prognostic factor (IGPF) signature was established by the RF algorithm, which displayed an enhanced prediction performance compared with the PRIF model alone (with average AUCs in the test dataset for 1-, 3-, and 5-year survival of 0.84 vs. 0.81, 0.81 vs. 0.74, and 0.75 vs. 0.68). The results suggested that the overall prognosis assessment may be credited to the involvement of imaging-genomics prognostic factors for ccRCC patients.

#### Metastasis

To assess the ability of a model constructed by integrating radiomics, genomics, and clinical features to predict the metastasis of colorectal cancer (CRC) patients, Liu et al. [80] retrospectively analysed 134 patients (primary cohort: 62, validation set: 28, independent test set: 44) clinicopathologically diagnosed with CRC and developed a multiscale preoperative model through multivariable logistic regression analysis for metastasis prediction, incorporating the above three types of features. Sixteen radiomics features and the expression levels of four identified genes were included in the multiscale nomogram model, which exhibited outstanding prediction performance, with AUCs of 0.981 in the primary dataset (95% CI 0.953–1.000), 0.822 in the validation dataset (95% CI 0.635–1.000), and 0.752 in the independent test dataset (95% CI 0.608–0.896), showing great potential to be applied clinically to assist in individualized preoperative metastasis evaluation in patients with CRC.

### Predicting treatment responses

It is difficult to predict patient response to treatment on account of the heterogeneity of solid tumours, the absence of consistent and accurate biomarkers, and a vague understanding of resistance mechanisms. The approach of radiogenomics has shed light on addressing these challenges through the untapped and abundant resource of imaging data [91]. Aiming to predict the response to chemotherapy and metastasis probability, Kim et al. [92] recruited 73 paediatric osteosarcoma patients and constructed a prediction model using an ML algorithm. The model combined the gene expression data of KI67 and EZRIN with image texture features extracted from fluorodeoxyglucose positron emission tomography/computed tomography (18F-FDG PET/CT) images. The results showed that the best test accuracy and AUC for predicting chemotherapy response with KI67 and EZRIN were 0.85 and 0.89, respectively. In recent research for predicting platinum resistance of ovarian cancer (OC), Yi et al. [93] generated an ML model incorporating radiomics data based on pretreatment CT images, clinicopathological data, and genomic data of single-nucleotide polymorphisms (SNPs) of human sulfatase 1 (SULF1). This combined model showed better classification efficiency, high calibration, and promising clinical utility, with AUC values of 0.993 (95% CI 0.83–0.98) in the training dataset (n = 71) and 0.967 (95% CI 0.83–0.98) in the validation dataset (n = 31).

### Predicting molecular characteristics

Through the combination of genomics data and CT radiomics features, it is likely feasible to predict the molecular characteristics of patients with cancer. In research on ccRCC, Zeng et al. [77] applied ML algorithms to predict gene mutations and different mRNA-based molecular subtypes according to radiomics features. The researchers collected the gene expression information of 207 ccRCC patients from TCGA and corresponding contrast-enhanced CT images from TCIA. The results proved excellent performance in detecting mutations of VHL (AUC: 0.971), PBRM1 (AUC: 0.972), BAP1 (AUC: 0.955), and SETD2 (AUC: 0.949), as well as the molecular subtypes, specifically m1 (AUC: 0.973), m2 (AUC: 0.968), m3 (AUC: 0.961), and m4 (AUC: 0.953), which were validated externally in another population containing 175 patients with ccRCC from West China Hospital. The study illustrated that integrative analysis of radiogenomics might be a practical approach to predict gene mutations or molecular characteristics in ccRCC patients, indicating a fast and noninvasive alternative to genetic testing.

Another multi-institutional retrospective study conducted by Park et al. [94] showed that preoperative MR imaging features may identify isocitrate dehydrogenase (IDH) wild-type lower-grade gliomas that have consistent molecular features as glioblastoma. The molecular features specifically included amplification of epidermal growth factor receptor (EGFR) or telomerase reverse transcriptase (TERT) promoter mutation. The research enrolled 64 patients in the training cohort and 57 patients in the validation cohort who were clinicopathologically diagnosed with IDH wild-type lower-grade gliomas. In the external test dataset, a model integrating both radiomic features and Visually AcceSAble Rembrandt Images revealed superior predictive performance (AUC of 0.854) than either that merely with clinical factors or Visually AcceSAble Rembrandt Images (AUCs of 0.514 and 0.648, respectively; P < 0.001, both). The predictive model showed the best performance when clinical features were added (AUCs of 0.514 vs. 0.863, P < 0.001).

Radiomics analysis is also capable of helping with the establishment of a molecular typing system. By performing quantitative radiomics analysis, Li et al. [95] demonstrated that MR image-based tumour phenotypes could predict the molecular classification of invasive breast cancers. The results revealed that the tumour phenotypes extracted by the computer allowed the distinction between molecular prognostic indicators, with AUC values of 0.89, 0.69, 0.65, and 0.67 when distinguishing ER + vs. ER − , PR + vs. PR − , HER2 + vs. HER2 − , and triple-negative vs. others, respectively.

## Challenges and future development

Despite the great potential shown by radiomics in clinical guidance, no developed radiomics signature is currently applied in the clinical field. There are still many challenges regarding the application of radiomics. A significant variation exists in every step during the whole analysis process derived from different sources, leading to the poor generalizability and reproducibility of the radiomics signatures previously published [96, 97]. For instance, the various settings in each stage of image acquisition, such as scanners, scanning techniques, and reconstruction parameters, might affect the extracted radiomics features. In recent research on renal clear cell carcinoma, Lu et al. [98] asserted that multiple uncontrolled confounders, including feature redundancy and image acquisition settings, especially tumour size and slice thickness, may bring about false or overvalued radiomics signatures. Considering the urgent need to standardize the radiomics process and accelerate its translation to clinical application, Lambin et al. [9] proposed the radiomics quality score (RQS) composed of sixteen criteria to evaluate both past and future radiomics studies, including well-documented image protocols that allow reproducibility or replicability, multiple segmentations, feature reduction or adjustment for multiple testing to decrease the overfitting risk, reporting discrimination statistics and calibration statistics, performing validation without retraining and adaptation of the cut-off value, etc. In summary, more standard guidelines are needed to normalize radiomics into reproducible and convincible analyses and to develop it as a mature field.

## Conclusions

The application of radiogenomics in oncology shows great prospects in precision medicine. The methodology of radiogenomics integrates large volumes of quantitative yet significant imaging phenotypes extracted from medical digital images and tumour genetic profiles derived from high-throughput sequencing, together with clinical-epidemiological data, into mathematical modelling. The amalgamation of radiomics and genomics provides an approach to better study the molecular mechanism of tumour pathogenesis, as well as new evidence-supporting strategies to identify the characteristics of cancer patients, make clinical decisions by predicting prognosis, and improve the development of individualized treatment guidance. However, many challenges hinder the application of radiomics in real clinical situations, such as the poor generalizability and reproducibility of the previously published radiomics models. Therefore, more standard guidelines are needed in future research to normalize radiomics into reproducible and convincible analysis and develop it as a mature discipline.

## Data Availability

Not applicable.

## Abbreviations

HGP: Human Genome Project
CT: Computed tomography
MRI: Magnetic resonance imaging
PET: Positron emission tomography
ROI: Region of interest
VOI: Volume of interest
CNN: Convolutional neural network
LASSO: Least absolute shrinkage and selection operator
PCA: Principal component analysis
GLM: Generalized linear model
ML: Machine learning
RF: Random forest
LR: Logistic regression
SVM: Support vector machine
ROC: Receiver operating characteristic
AUC: Area under the curve
DCA: Decision curve analysis
OS: Overall survival
PFS: Progression-free survival
DFS: Disease-free survival
ccRCC: Clear cell renal cell carcinoma
NSCLC: Non-small cell lung cancer
MB: Medulloblastoma
ESCC: Oesophageal squamous cell carcinoma
CRC: Colorectal cancer
OC: Ovarian cancer
BC: Breast cancer
